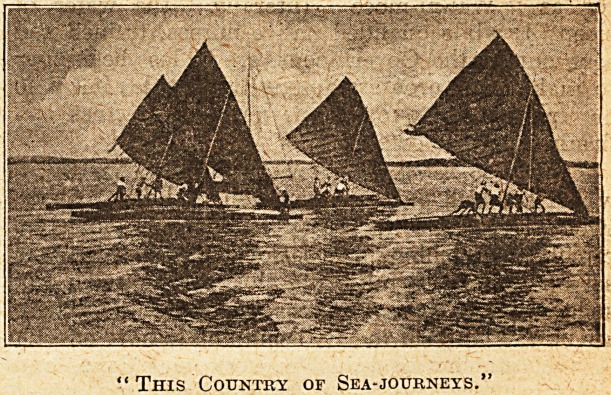# Curiosities of Native Treatment in Fiji

**Published:** 1919-03-08

**Authors:** T. R. St.-Johnston

**Affiliations:** Colonial Civil Service.


### CURIOSITIES OF NATIVE TREATMENT IN FIJI.

IV. By T. R. ST.-JOHNSTON, M.R.C.S., L.R.C.P., Colonial Civil Service.

After the child is born the mother, if of high rank, does not appear outside the house or follow any occupation for at least ten days, often a month. This is not because of any fear for her own health, but because the baby may not run any risks, a chief's child, especially if a male, being an item of very considerable importance. And whether chief or commoner the mother will not venture to do any sea-bathing or shore-fishing, as sea-water is supposed to have a very injurious

A Feast : Turtle Inverted and Yams.

effect upon her. She also partakes only of a vegetable diet during this period, as this is said to promote lactation.

If the breast is dry there are various ways to bring the milk, some of which are really quite useful. Firstly, the breasts are well oiled with scented coconut oil, and gentle but expert massage is applied to them. Then they are carefully dried and steamed or fomented with hot cloths, and finally a poultice of macerated leaves, sometimes of the abortifacient plant, "Ti Kula," is placed on them. Probably the idea of the latter is an instance of the magic that "like produces like." If with all these endeavours the breasts still refuse their function, a wet-nurse is called in, but usually these are very reluctant foster-mothers, unless actually ordered by a chief, and the infant therefore stands a poor chance of life; for only very close relatives, such as the "tina lailai" (aunt, literally "little mother"), take sufficient interest in the child to render any help; and no question of payment in the way of presents can overcome this reluctance. When all else fails, Lau Islanders, like the Tongans, chew dalo, yam, and other starchy vegetables, and give the chewed result to the baby to assimilate; which is not altogether a bad idea, as the mother's saliva does the necessary conversion of starch into maltose, and prevents the diarrhœa that would otherwise ensue. But now that tuberculosis is appearing among them this is not a good practice to encourage.

On the tenth night there is a ceremonial feast, and again on the hundredth night, and there is even a feast when the baby's first hair-cutting is performed! For these grown-up children of the South Seas delight in ceremonial whenever possible, and a feast is the natural adjunct of most ceremonies.

Lactation, in a country where a cow, goat, or other milk-giving animal was unknown before the white man introduced it, is naturally very prolonged, and the infant continues to draw its mother's milk until sturdy and able to run about. I once saw in the Sigatoka River district a big healthy boy of five (who was, in a childish way, chopping brush firewood for the family kitchen) drop his large knife, and, running across to his mother, take a pull at her breast; but this was, of course, an extreme case.

The children when first born are not usually big, which is surprising in such a fine muscular race as the Fijians. It is only after puberty that they suddenly develop, and they increase at a rapid rate. This comparatively small size of the baby in proportion to the mother is another reason why the births are fairly easy with these people, though there are, of course, exceptions to the general rule, and, as may be imagined, the obtaining of skilled assistance in time to prevent a fatality is not an easy matter in this country of sea-journeys and mountain tracks. I happened on one occasion to be about two days' journey up the higher reaches of a river when it was reported to me that a primipara had been in labour for some time in a neighbouring village, and could not be delivered. I had nothing with me but a pocket-knife, as my servant

"This Country of Sea-journeys."

had lost my little surgical case the day before; and on examining the woman I found that it was a case of locked twins, quite impossible to manipulate in any way, and pulseless, and apparently now dead. Eventually I managed to get hold of a short length of fencing-wire and an old pair of 8-inch scissors with about an inch broken off one blade. I boiled these and my knife in a kettle (even that was a lucky find in this primitive village of grass huts), and after many attempts and some laceration managed

Previous articles appeared in the "Hospital" of January 18, February 8 and 22, page 445.

**Curiosities of Native Treatment in Fiji**—(*cont.*).

to hook down a neck, decapitate a head, and deliver the bodies of both children. I took the mother in a leaking punt down to a hospital at the river mouth, and to my surprise she made an uninterrupted recovery.

On one occasion I received a message from another island that a baby with five arms was impacted, and that the mother was dying of the obstruction! Getting across the same evening in a small sailing-boat I found a typical instance of how native rumours and exaggerations get spread. It appeared that it was a shoulder and arm presentation, and the first thing noticed earlier in the day had been the five fingers of a hand appearing. A messenger had at once been sent off with a verbal message, which by the time it reached me had extended to five arms! I found the mother sitting on the floor on some blood-stained mats, with the pulseless protruding hand well exposed on this very septic resting-place. After washing it and the parts well with antiseptics, I managed to replace it and do a version, the mother in this case also making a good recovery, with no sign of fever. About the same time I heard of a baby being born "with the head of a turtle," this being undoubtedly another instance of a mouth-to-mouth native yarn. But unusual developments are really rare, and are in fact attributed impartially to a "tevoro" (a devil) or to adultery; though I have triplets recorded in my notes as occurring twice in the same month, and these were considered as quite proper and decent, being suitable cases for an application for remission of taxes!

Chloroform is now becoming understood and appreciated by native women on those occasions when they *do* have a hard time, and is readily asked for as "Wai-ni-moce" (literally "the water of sleep"); while qualified native nurses, trained and sent out by the Government, are at last beginning to make some progress among the people; but it is an uphill task to overcome native prejudices and firmly-fixed customs.

Finally, a word should be said about abortions, which are still only too common in Fiji. About twenty years ago the Fijian race was found to be rapidly diminishing, and a Royal Commission was set up to inquire fully into this matter and make suggestions to stem the receding current. (These are now, I am glad to say, beginning to have effect.) A vast amount of valuable work was done, and many causes were found for the decrease, one of the chief ones being the large number of abortions, both involuntary and pathological as well as voluntary and criminal.

Abortions due to disease, in a people among whom syphilis is unknown, are yet not infrequent, and are often due to the allied disorder frambœsia or yaws, which is almost universally prevalent throughout the country. Although congenital yaws is not found, yet the mothers are often sufferers from large tertiary manifestations at the time of pregnancy, and there is no doubt that this must have a bad effect on the enlarged uterus and vascular system generally. Bouts of filarial fever also will doubtlessly affect the fœtus, and there are very few Fijians who are altogether exempt from this.

But when we turn to deliberate abortion-procuring we find that the Fijians as a race have been from time immemorial adepts at the practice, and the same "yalewa vuku," or midwife, who assists in the delivery of a healthy living child will just as cheerfully lend her services to the expulsion of an undesired fœtus. Although every endeavour is made to deal with the offence as a criminal one, it is for obvious reasons very seldom that a case can be proved, and there is no doubt that it still goes on, especially as the native women are becoming under British government more relaxed in their morals, owing to their freedom from fear of the old death penalty for adultery. Naturally, their safety from such a violent ending is the more important thing, and I only mention this to show that the upsetting of native customs by civilisation does not always bring unmixed blessings in its train.

The old women are wont to bring about an abortion either by the insertion of a smooth prepared stick ("losi-losi-sau") in the uterus, or by manipulation under water at the "testing for pregnancy" that I mentioned earlier, or by the giving of certain decoctions by the mouth. There are many plants that great faith is held in as abortifacients, but, naturally, these secrets are carefully guarded, and it is only rarely that one can get on the track of anything of this sort. I remember once going suddenly into the hut of an old woman who had been under suspicion for some time, and finding in the eaves a mysterious package of crushed leaves done up in a sheet of fibre torn from the stem of a coconut tree; but she swore that it was only native medicine for her rheumatism, and there was no proof of any definite case to tax her with.

A white man does occasionally get hold of this knowledge if he has intermarried with and lived among the people, and from such men one knows of certain of the abortifacient plants. The most esteemed of all is undoubtedly the "Ti Kula" (*Dracæna ferrea*), but others extensively used are "Kala Kala Waisoni" (*Hibiscus diversifolius*), "Siti" (*Grewia prunifolia*), "Wakiwaki" (*Hibiscus moschatus*), and "Wavuti" (*Pharbitis insularis*). Several of these are also purgatives, and the native well understands that a violent purge may at a delicate period tend to aid a miscarriage.

The most extraordinary miscarriage I remember was one reported to me by a Government native doctor attached to one of the hospitals in my district. He found that the patient had produced what he described as a large red mass of the consistency of a turtle's flipper, and that this was firmly believed by all the townspeople and the woman herself to be the offspring of Daucina (an ancient god or devil), who had attacked her in an unconscious state while sleeping under the same mosquito curtain as her husband. When I afterwards saw the woman she admitted that it was unusual, not the fact that Daucina was the parent, but that his offspring had in other known instances been always hard stones!

(*Concluded.*)

## Figures and Tables

**Figure f1:**
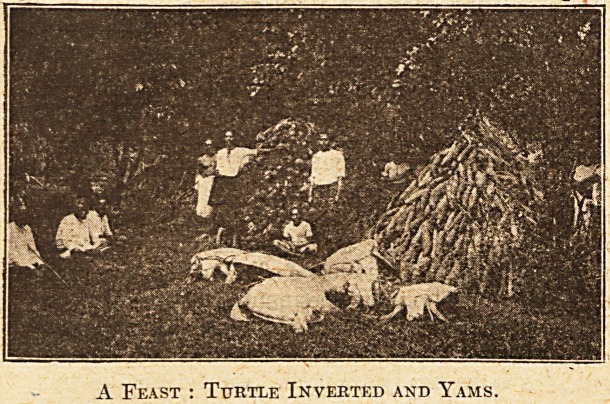


**Figure f2:**